# Distinct Co-occurrence Relationships and Assembly Processes of Active Methane-Oxidizing Bacterial Communities Between Paddy and Natural Wetlands of Northeast China

**DOI:** 10.3389/fmicb.2022.809074

**Published:** 2022-01-26

**Authors:** Xu Liu, Yu Shi, Teng Yang, Gui-Feng Gao, Liyan Zhang, Ruoyu Xu, Chenxin Li, Ruiyang Liu, Junjie Liu, Haiyan Chu

**Affiliations:** ^1^State Key Laboratory of Soil and Sustainable Agriculture, Institute of Soil Science, Chinese Academy of Sciences, Nanjing, China; ^2^University of Chinese Academy of Sciences, Beijing, China; ^3^State Key Laboratory of Crop Stress Adaptation and Improvement, School of Life Sciences, Henan University, Kaifeng, China; ^4^Key Laboratory of Integrated Regulation and Resource Development on Shallow Lake of Ministry of Education, College of Environment, Hohai University, Nanjing, China; ^5^High School Affiliated to Nanjing Normal University, Nanjing, China; ^6^Key Laboratory of Mollisols Agroecology, Northeast Institute of Geography and Agroecology, Chinese Academy of Sciences, Harbin, China

**Keywords:** CH_4_ oxidation, DNA-SIP, methane-oxidizing bacterial communities, co-occurrence relationships, assembly processes

## Abstract

Studies of methane-oxidizing bacteria are updating our views of their composition and function in paddy and natural wetlands. However, few studies have characterized differences in the methane-oxidizing bacterial communities between paddy and natural wetlands. Here, we conducted a ^13^C stable isotope-probing experiment and high-throughput sequencing to determine the structure profiling, co-occurrence relationships, and assembly processes of methanotrophic communities in four wetlands of Northeast China. There was a clear difference in community structure between paddy and natural wetlands. LEfSe analyses revealed that *Methylobacter*, *FWs*, and *Methylosinus* were enriched in natural wetlands, while *Methylosarcina* were prevailing in paddy, all identified as indicative methanotrophs. We observed distinct co-occurrence relationships between paddy and natural wetlands: more robust and complex connections in natural wetlands than paddy wetlands. Furthermore, the relative importance of stochastic processes was greater than that of deterministic processes, as stochastic processes explained >50% of the variation in communities. These results demonstrated that the co-occurrence relationships and assembly processes of active methanotrophic communities in paddy and natural wetlands were distinct. Overall, the results of this study enhance our understanding of the communities of methane-oxidizing bacteria in paddy and natural wetlands of Northeast China.

## Introduction

Wetland ecosystem is one of the largest terrestrial sources of methane (CH_4_) emissions. Paddy wetlands account for approximately 24 to 40 Tg CH_4_ yr^−1^ and natural wetlands account for 100 to 183 Tg CH_4_ yr^−1^ (Saunois et al., 2020), totally contributing about one-third of global CH_4_ emissions (Bousquet et al., 2006). More than 80% of CH_4_ produced is oxidized by methane-oxidizing bacteria (MOB or methanotrophs) before being released into the atmosphere (Mer and Roger, 2001). Wetlands soils are dynamic systems characterized by high methanotrophic activity at the aerobic–anaerobic interface; microbial guilds involved in CH_4_ consumption in such soils in paddy and natural wetlands have received increased interest from researchers (Chowdhury and Dick, 2013; Kirschke et al., 2013). For example, Shiau et al. (2018) found that the *Methylocystis*-affiliated type II genotype is the predominant methanotroph in paddy fields at the regional scale of ~400 km. Yun et al. (2012) suggested that CH_4_ consumption in Zoige natural wetland was driven by both type I and type II methanotrophs. Although several community studies of methanotrophs have been conducted, comparative studies of the structure of methanotrophic communities in paddy and natural wetlands are lacking.

Biotic interactions play a critical role in shaping the co-occurrence patterns of microbial guilds, and different co-occurrence patterns of methanotrophic communities are supposed to be correlated with essential ecological processes, such as polymer breakdown, syntrophy, and fermentation (Peng et al., 2018; Zhang et al., 2020). Microbial guilds compete with syntrophic partners to obtain carbon and ATP (Conrad, 2009). These partnerships are shaped by metabolic interactions as well as the degree of habitat niche overlap (Faust and Raes, 2012). However, detailed information on interspecific and intraspecific associations in this field based on empirical laboratory studies is difficult to obtain, especially for complex and diverse microbial communities. Inferring co-occurrence networks based on ecological data can provide key insights into co-occurrence relationships, the links among communities, and the functional potential of communities (Nemergut et al., 2013; Röttjers and Faust, 2018; Yang, 2021). For example, Li et al. (2021) explored the coexistence patterns of soil methanogens and revealed that the complex interactions are closely tied to CH_4_ generation. Co-occurrence relationships are distinct from ecological interactions, and exploration of the former could provide new insights into the structure and functional potential of methanotrophic communities in paddy and natural wetlands.

The role of assembly mechanisms in determining the structure and function of methanotrophic communities has been a major focus of previous studies (McCalley et al., 2014; Hines, 2019). Comparison of intra- and inter-group phylogenetic distances with null models has provided new insights into assembly mechanisms, and this work has emphasized the importance of obtaining phylogenetic information for describing and predicting the structure and function of microbial communities (Stegen et al., 2013; Ning et al., 2020). Phylogenetic clustering and overdispersion compared with null models indicate the emphatic importance of deterministic processes in shaping community structure, representing variable and homogeneous selection, respectively (Emerson and Gillespie, 2008; Vellend, 2010; Weiher et al., 2011). Phylogenetic clustering could lead to the analogy of functional genotypes, whereas phylogenetic overdispersion indicates that diverse functional guilds are phylogenetically more distantly related to each other (Wang et al., 2012; Mittelbach and Schemske, 2015). For example, Fan et al. (2019) found that long-term fertilization shifts nitrogen fixers from being phylogenetically clustered to being phylogenetically overdispersed, which leads to the emergence of more phylogenetically diverse diazotrophic communities. In addition to deterministic processes, community assembly is mediated by stochastic processes, such as ecological drift and dispersal (Stegen et al., 2015). Stochastic processes play an important role in driving microbial community patterns in multiple ecosystems (Jiao et al., 2019). Generally, study of the assembly processes of methanotrophs can enhance our understanding of MOB communities and their functional potentials in paddy and natural wetlands.

Stable isotope probing (SIP) has been used in many ecological studies and is effective for studying the metabolic activities of methanotrophs (Dumont et al., 2011; Ho et al., 2013). A direct link between CH_4_-uptake activity and methanotrophic taxa has been established using SIP and analysis of DNA markers specific to methanotrophs by feeding them with ^13^C-labeled CH_4_ (He et al., 2019, 2021; Sultana et al., 2019; Zhang et al., 2020). Here, we conducted a DNA-SIP experiment combined with high-throughput sequencing and multiple bioinformatics methods in four typical wetlands of Northeast China to study differences in methanotrophic communities between paddy and natural wetlands. Specifically, the aims of this study were to (i) identify differentially abundant and indicative methanotrophs in paddy and natural wetlands; (ii) characterize differences in the co-occurrence relationships and assembly processes of methanotrophic communities in paddy and natural wetlands; and (iii) explore the links between methanotrophic communities and functional potentials. We hypothesized that not only would the community compositions of active methanotrophs be distinct between paddy and natural wetlands, the co-occurrence relationships and assembly processes would be also different between paddy and natural wetlands. Overall, the results of our study provide new insights that could be used to aid the management of wetland ecosystems and enhance CH_4_ consumption.

## Materials and Methods

### Study Site and Sampling

**Figure 1:**
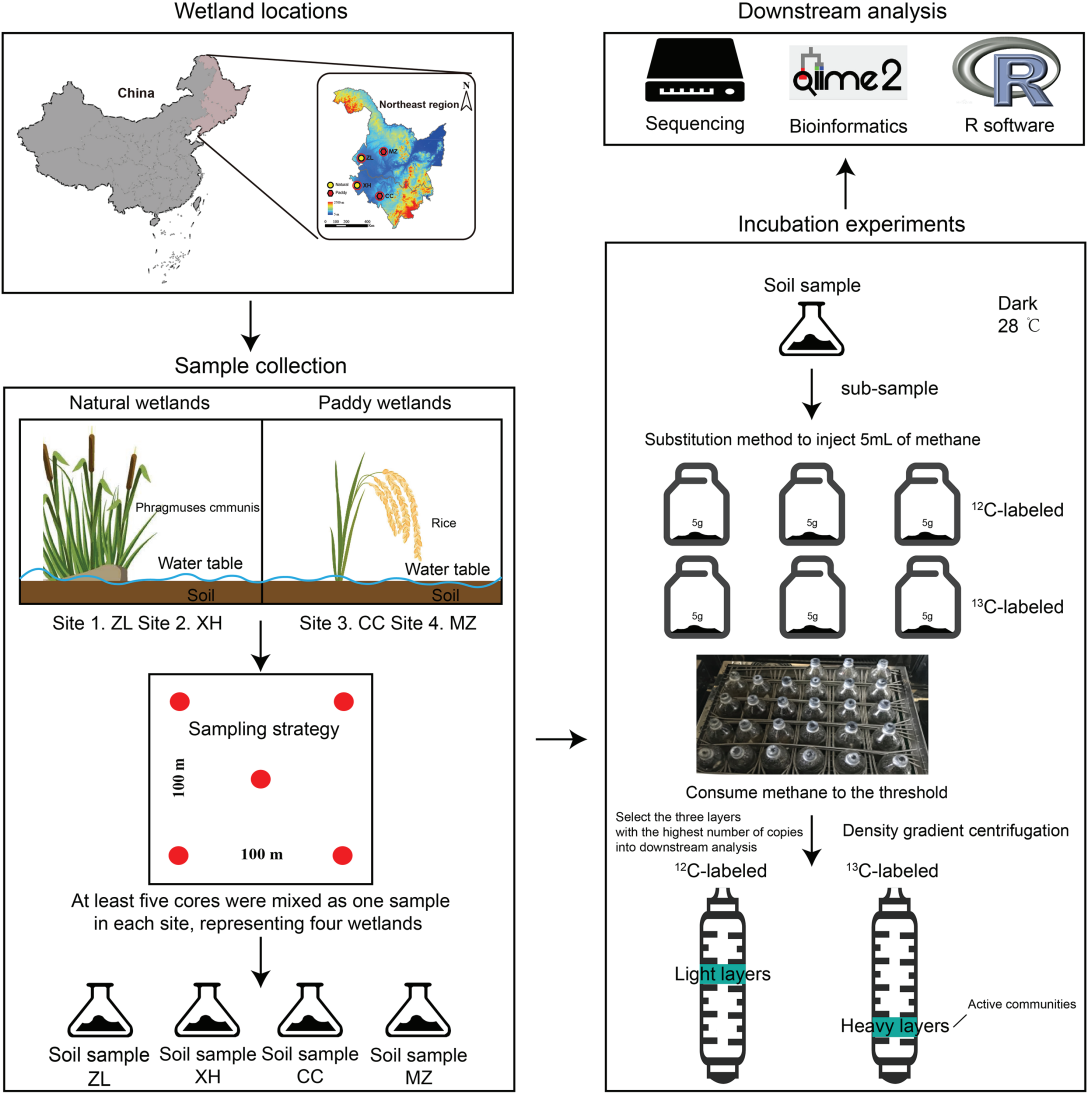
A pipeline of the study on the differences in active methanotrophic communities between paddy and natural wetlands. CC on behalf of Changchun paddy wetland, MZ on behalf of Minzhu paddy wetland, XH on behalf of Xianghai natural wetland, and ZL on behalf of Zhalong natural wetland.

Northeast China has a low mean annual temperature and is rich in soil carbon stocks; thus, the potential of carbon turnover is enormous (Tang et al., 2018). Paddy and natural wetlands are widespread in this region, accounting for ~16% of the total wetland area in China (Ding and Cai, 2007). Natural wetland soils were collected from two Chinese national wetland parks in Northeast China: Zhalong (ZL) and Xianghai (XH). Paddy wetland soils were collected after the rice harvest in Changchun (CC) and Minzhu (MZ) in Northeast China. In each wetland, at least five soil cores were collected from depths of 0 to 20 cm. The cores were taken approximately 100 m apart and mixed to form one soil sample for each wetland. Visible roots and residues were eliminated from the soil samples. Samples were sieved through 2-mm mesh after natural withering and stored at 4°C before microcosm experiments. Detailed information on the study sites and sampling is provided in Figure 1 (wetland locations and sample collection) and Supplementary Table S1.

### DNA-SIP Microcosms

The four soil samples were treated with ^12^C-labeled and ^13^C-labeled CH_4_ in triplicate in microcosms (4 sites × 2 treatments × 3 replicates = 24 total experimental units; Figure 1). For each microcosm, 5.0 g of soil (dry weight) was incubated at approximately 60% of the maximum water-holding capacity and 28°C in the dark in a 120-ml serum bottle sealed with a butyl stopper to determine the depletion of CH_4_ in soils (Jia et al., 2019). For all incubations, 5.0 ml of the headspace air in the bottles was replaced with the same amount of ^12^CH_4_ and ^13^CH_4_ gas, which resulted in an initial CH_4_ mixing ratio of approximately 40,000 ppmv in the headspace (Sultana et al., 2019). The CH_4_ gas was >99% ^13^C-atom pure. Gas chromatography was used to quantify the headspace CH_4_ concentrations at two-day regular intervals (Shimadzu GC12-A, Japan). When more than 90% of CH_4_ in the soil was consumed, soil samples were collected and separated into two subsamples for DNA extraction and long-term preservation. Methane oxidation potential (MOP) was calculated using the formula $\mathrm{MOPs}=\frac{\left(C1-C2\right)*120*0.001*16*301}{22.4*273*T}$, where *C*1 and *C*2 represent the CH_4_ concentration at the finishing time and starting time, respectively, and *T* stands for the temperature of the experiment.

### DNA Extraction and SIP Gradient Fractionation

For the molecular survey of methanotrophic communities, 0.5 g of incubated soil of each microcosm was used for total DNA extraction with a FastDNA^®^ Spin Kit (MP Biomedicals, Santa Ana, CA) per the manufacturer’s instructions. The extracted DNA was dissolved in 70 μl of TE buffer. The quality and concentration of extracted DNA were quantified by NanoDrop1000 spectrophotometer (Thermo Fisher). When A260/A280 > 1.8 and A260/A230 > 2, the extracted DNA was considered of high quality and free from protein or RNA contamination. Besides, the samples were filtered with too low DNA concentration (<20 μg/ml). DNA samples that did not meet the above criteria were re-extracted. The qualified DNA was separated into two subsamples for high-speed buoyancy density centrifugation and long-term preservation.

To separate the different weights of labeled (^13^CH_4_) and unlabeled (^12^CH_4_) DNA, density gradient centrifugation of all extracted DNA was conducted. Soil DNA samples were collected from all CH_4_-incubated microcosms (Lu and Jia, 2013). After adding CsCl solution into 5-ml sterile tubes with 2.0 μg of DNA, the mixed solution was adjusted with gradient buffer to a final density of 1.725 g/ml. The adjusted solution was then centrifuged in an ultracentrifuge (Beckman Coulter, Palo Alto, CA) at 177,000 g for 44 h at 20°C. DNA fractionations were carried out by the substitute method, wherein the gradient medium was displaced with sterile water from the top of the centrifuge tubes at a precisely controlled flow rate of 0.38 ml/min. Fifteen fractions per tube were collected and weighed on a 10,000 ppm balance. Polyethylene glycol 6000 (PEG6000) was used to precipitate the fractionated DNA from the calcium chloride medium, which was subsequently purified with 70% ethanol and dissolved in 30 μl of TE buffer for further amplification and sequencing.

### Real-Time Quantitative PCR of *pmoA* Genes

Real-time quantitative PCR (qPCR) analysis was conducted using the CFX96 optical real-time detection system to determine the efficiency at which ^13^C was incorporated into genomic DNA from methanotrophic communities (Bio-Rad, United States). The primers A189F (5′-GGNGACTGGGACTTCTGG-3′) and mb661r (5′-CCGGMGCAACGTCYTTACC-3′) were used to quantify the number of *pmoA* gene copies (Costello and Lidstrom, 1999). Reactions were performed in triplicate for each fractionated DNA sample. Reaction conditions were as follows: 95°C for 60 s, followed by 39 cycles at 95°C for 30 s, 55°C for 30 s, and 72°C for 30 s. Melting curve analysis was conducted by increasing the temperature from 65°C to 95°C in 0.1°C per second increments with continuous fluorescence acquisition. Individual standards were obtained from a 10-fold dilution series of plasmids containing a single *pmoA* gene fragment. Amplification efficiencies ranged from 91.6 to 96.7%, with *R*^2^ values of 0.995–1.

### Amplification and Sequencing of *pmoA* Gene Sequences

Three representative fractionations with a high copy number of the *pmoA* gene of each ^13^C-labeled microcosm (heavy density of 1.7227–1.7422 g/ml) were selected according to the qPCR results. The A189F/mb661r primer set was used to amplify the *pmoA* genes, as it could retrieve the highest diversity of soil methanotrophs (Costello and Lidstrom, 1999). PCR conditions and procedures are commonly used to sequence *pmoA* functional genes (Zhang et al., 2020). Before sequencing, all PCR products were standardized to equimolar levels, and high-throughput sequencing was carried out using an Illumina MiSeq PE300 platform (Illumina, Inc., San Diego, CA, United States). All sequencing data in this study were deposited in the NCBI Sequence Read Archive (SRA) under the BioProject accession number PRJNA662020.

The raw data were analyzed using the QIIME pipeline (version 1.9.1; Caporaso et al., 2010). The raw reads were denoised to eliminate low-quality reads (length less than 200 bp or average quality score less than 29) to obtain high-quality sequences. The uchime3-denovo method in VSEARCH was used to detect chimeras (Rognes et al., 2016). Insertions and deletions causing the frameshifts in *pmoA* gene sequences were corrected using FrameBot^1^ (Cole et al., 2014). The high-quality sequences were clustered into different operational taxonomic units (OTUs) by UCLUST (Edgar, 2010) based on a 93% similarity threshold of *pmoA* gene sequences (Degelmann et al., 2010). The PyNAST method was used to align representative sequences (DeSantis et al., 2006). The RDP-classifier (Cole et al., 2014) was used to determine the taxonomic identity of the OTUs, which was based on a database including 6,628 *pmoA* and *pmoA*-related sequences from pure culture methanotrophs and uncultured methanotrophic ecotypes (Dumont et al., 2014). After filtering low-quality reads and rarefying samples to equal sequencing depth, we obtained a total of approximately 115,754 quality-filtered, chimera-free, and frameshift-free sequences with an average of 1,172 methanotroph *pmoA* gene reads (the minimum number of sequences of all samples) for downstream analysis.

### Co-occurrence Relationship Inference

Co-occurrence relationship inference was conducted at the OTU level *via* the SPIEC-EASI network method with the function *spiec.easi* in the spieceasi package (Kurtz et al., 2015). We focused on the dominant methanotrophs with filtered frequency (top 10% of relative abundance of active methanotrophs) and occurrence (>25% of all samples) across all wetland soils. Based on the filtered conditions, we constructed the total co-occurrence network involving 36 samples and then extracted the sub-network according to two types of wetlands. The generated network results were imported into Gephi software to determine the topological properties. Furthermore, we used within-module (z-score) and among-module (c-score) connectivity as a topological indicator to classify nodes as network hubs (z-score > 2.5; c-score > 0.6), module hubs (z-score > 2.5; c-score < 0.6), connectors (z-score < 2.5; c-score > 0.6), and peripherals (z-score < 2.5; c-score < 0.6; Shi et al., 2020). Module abundance in each network was calculated by averaging the standardized relative abundances (z-score) of the nodes that belonged to the specific modules. We used ForceAtlas 2 algorithms to display the visualization plot of the co-occurrence network in the Gephi platform.^2^

### Quantification of Assembly Processes

Standardized mean pairwise distance (MPD) was calculated to determine the phylogenetic distance among methanotrophic communities using the picante package. This reveals the degree of dispersion of lineages within a community (Kembel et al., 2010). MPD values < −2 indicate phylogenetic clustering; MPD values > 2 indicate phylogenetic overdispersion; and MPD values between −2 and 2 indicate phylogenetic stochasticity.

The β-nearest taxon index (βNTI) and Bray–Curtis-based Raup–Crick metrics (RC_bray_) were used to indicate the relative contributions of assembly processes, including deterministic processes (homogeneous selection and variable selection) and stochastic processes (homogenizing dispersal and dispersal limitation coupled with undominated processes). βNTI measures the deviation of observed β-mean nearest taxon distance (βMNTD) from the expected βMNTD in the null model. βNTI < −2 indicates homogeneous selection (HS), whereas βNTI > 2 indicates variable selection (VS). When |βNTI| < 2 and RC_bray_ > 0.95, dispersion limitation (DL) plays a dominant role. When |βNTI| < 2 and RC_bray_ < −0.95, homogenizing dispersal (HD) plays a dominant role. When |βNTI| < 2 and |RC_bray_| < 0.95, no single process plays a dominant role (UD), and this is also known as drift (Stegen et al., 2013).

### Statistical Analysis and Visualization

IBM SPSS Statistics 23.0 was used to perform analysis of variance, correlation analysis, and non-parametric difference tests (SPSS Inc., Cary, NC, United States). *Post hoc* Turkey’s tests were conducted for multiple comparisons using the EasyStats package in R (version 3.6.1).^3^ Differential abundance was determined using the DESeq2 package (Love et al., 2014). Principal coordinate analysis (PCoA) was conducted using the vegan package. Analysis of similarity (ANOSIM) in the vegan package was used to determine differences in community composition. Linear discriminant analysis effect size (LEfSe) analysis was used to identify the taxa that were differentially abundant and indicative in paddy and natural wetlands and was performed using the lefse package (Segata et al., 2011). Representative OTUs of methanotrophs were visualized using the iTOL website (Letunic and Bork, 2016).

Threshold indicator taxa analysis was conducted to identify indicator taxa using the TITAN2 package (Baker and King, 2010). This analysis uses standardized scores (Z-scores) to detect the MOPs exceeding thresholds based on their frequency and occurrence patterns. The z-scores were derived from normalizing indicator value scores (IndVals) with random permutations to determine the potential change. Responses of methanotrophs were standardized to the mean and standard deviation of permuted samples. Thus, the sum of the z-scores reflects the magnitude of potential change. TITAN differentiates taxa with positive (Z^+^) and negative (Z^−^) values; Z^+^ taxa increase in frequency and abundance after the change point, and Z^−^ taxa show the opposite pattern.

## Results

### Methane Oxidation Potential

**Figure 2:**
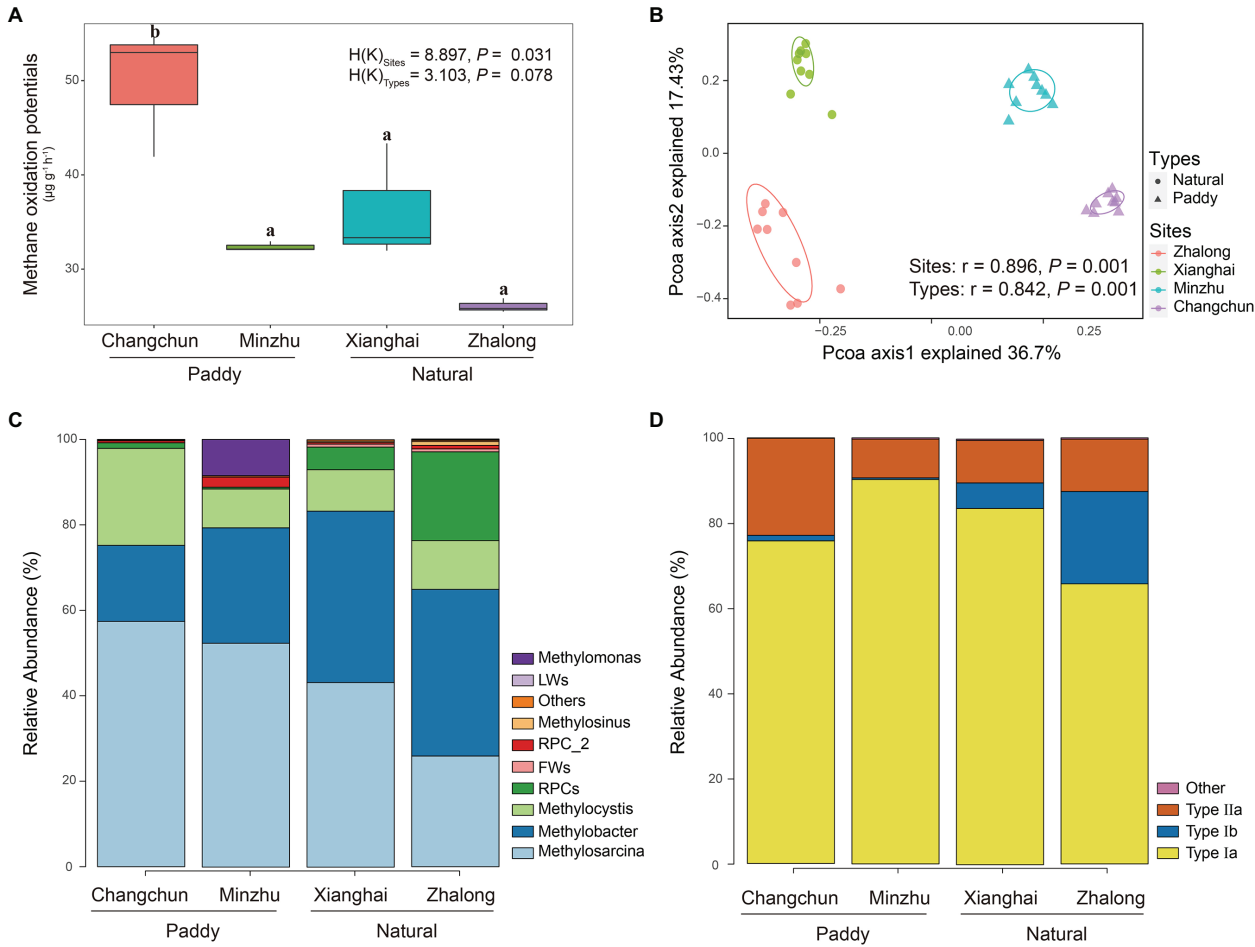
**(A)** Methane oxidation potentials at the CH_4_ incubations of four wetland soils. Boxes with different lowercase letters represent significant differences (*p* < 0.05). **(B)** Principal coordinates analysis (PCoA) biplots of Bray–Curtis distances for the active methanotrophic communities of four wetland soils. **(C,D)** Relative abundance of active methanotrophs on the genus levels and methanotrophic category levels identified with *pmoA* gene sequencing in four wetland soils.

DNA-SIP microcosm experiments revealed that all soil samples from paddy and natural wetlands displayed intense CH_4_ consumption (Figure 2A). Assuming linear kinetics, MOP was highest in Changchun, followed by Xianghai, Minzhu, and Zhalong. No significant differences were observed in the MOP of all incubations between paddy and natural wetlands [H(K) = 3.103, *p* = 0.078], but significant differences in MOP were observed among the four wetland sites [H(K) = 8.897, *p* = 0.031].

### Community Profiling of MOB Communities

Quantitative results of *pmoA* gene copy numbers across the entire buoyant density gradient of the fractionated DNA samples showed that the relative abundances of *pmoA* gene ^13^C-labeled in heavy DNA fractions were significantly increased, compared to the background values from the same DNA fractions in the ^12^C-labeled control treatment (Supplementary Figure S1). High-throughput sequencing of the *pmoA* genes was performed on C-labeled samples from SIP microcosms. The community structure of ^12^C-labeled MOB is shown in Supplementary Figure S2 as background data. The subsequent analysis focused on the ^13^C-labeled MOB, which were considered active MOB communities.

**Figure 3:**
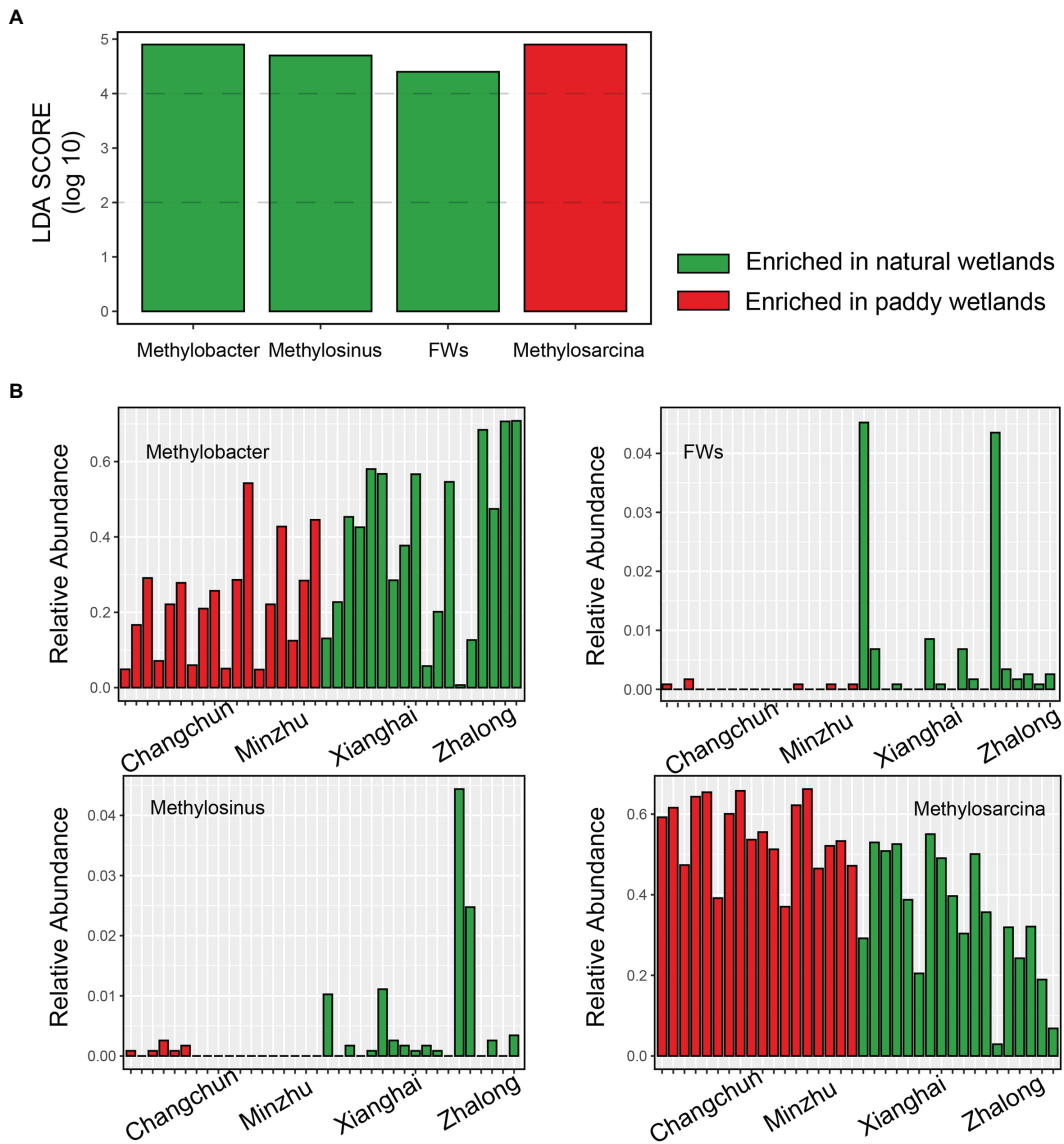
The upper barplot showed LDA score of the active methanotrophic communities between paddy and natural wetlands identified by Lefse analysis **(A)**. The following four histograms showed the relative abundance of indicative methanotrophs that make differences **(B)**.

Species richness and Faith’s phylogenetic diversity did not significantly differ among wetland sites (Supplementary Figure S3). No correlations were observed between MOPs and alpha diversity (Supplementary Table S2). The only correlation detected was a significant relationship between the relative abundance of *Methylosarcina* and MOP at the genus level (*r* = 0.734, *p* = 0.007; Supplementary Table S3). The PCoA plot revealed large differences in the community composition of the four wetland sites (ANOSIM *r* = 0.896, *p* = 0.001) and two wetland types (ANOSIM *r* = 0.842, *p* = 0.001), and these findings were consistent with the other dissimilarity distances (Figure 2B; Supplementary Table S4). The type Ia methanotroph *Methylosarcina* was the most common taxon in each incubation (Figures 2C,D); it was the most common in the CC incubations, accounting for ~57.4% of total sequences (~25.9% in ZL; ~43.2% in XH; and ~52.4% in MZ). *Methylobacter*-affiliated *pmoA* genotypes were also common in all the incubations (~39.0% in ZL; ~40.1% in XH; ~27.0% in MZ; and ~17.8% in CC). Type Ib methanotroph *RPCs* were only common in ZL (~22.7% in ZL; ~4.2% in XH; ~0.5% in MZ; and ~1.6% in CC), and the type II methanotroph *Methylocystis* was only common in CC (~11.4% in ZL; ~9.7% in XH; ~9.1% in MZ; and ~22.7% in CC). The type II methanotroph *Methylomonas* was common in MZ (~8.5%). These methanotrophs accounted for more than 90% of the total microbes. LEfSe analysis revealed that *Methylobacter*, *Methylosinus*, and *FWs*-affiliated clusters were enriched in natural wetlands, and *Methylosarcina* was more prevalent in paddy than in natural wetlands, these methanotrophs used to distinguish the MOB communities between paddy and natural wetlands as indicators (Figure 3).

In the TITAN2 analysis, the peaks Z^−^ and Z^+^ with the gradient of MOPs appeared around 13 and 30 μg g^−1^ h^−1^, respectively. In paddy wetlands, type I *Methylosarcina* and type II *Methylocystis* were Z^+^ taxa, which increased in frequency and occurrence as MOPs changed, whereas only type I genotypes (e.g., *Methylomonas*, *RPC_2*, *Methylosarcina*, and *Methylobacter*) were Z^−^ taxa (Supplementary Figure S4). Z^−^ and Z^+^ taxa showed opposite patterns in natural wetlands: Type I and type II genotypes were generally Z^−^ taxa, and type I genotypes were generally Z^+^ taxa (with the exception of 2 OTUs).

### Co-occurrence Relationships of MOB Communities

**Figure 4:**
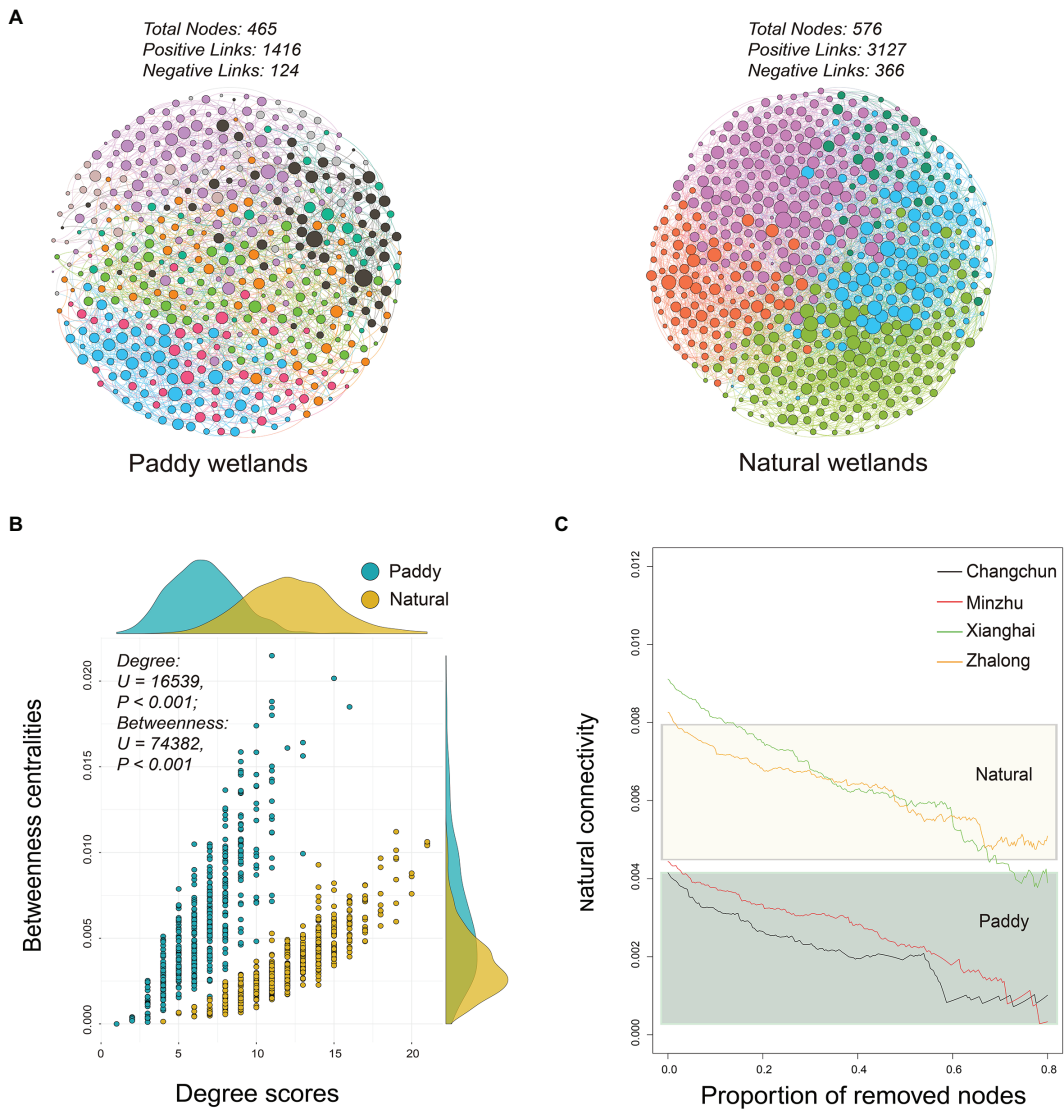
**(A)** Networks visualizing co-occurring phylotypes of active methanotrophs in paddy and natural wetlands. All co-occurrence networks were analyzed by Spiec-Easi method. Colorful nodes represented different ecological module affiliations of active methanotrophs and edges on behalf of potential ecological co-occurring relationships. **(B)** Relationships between degree scores and betweenness centralities in active methanotrophic co-occurrence networks of four wetland soils. The difference test was adopted by Mann–Whitney U test. **(C)** Network robustness analysis of active methanotrophic co-occurrence networks in four wetland soils, measured as natural connectivities with remained proportion of nodes.

**Figure 5:**
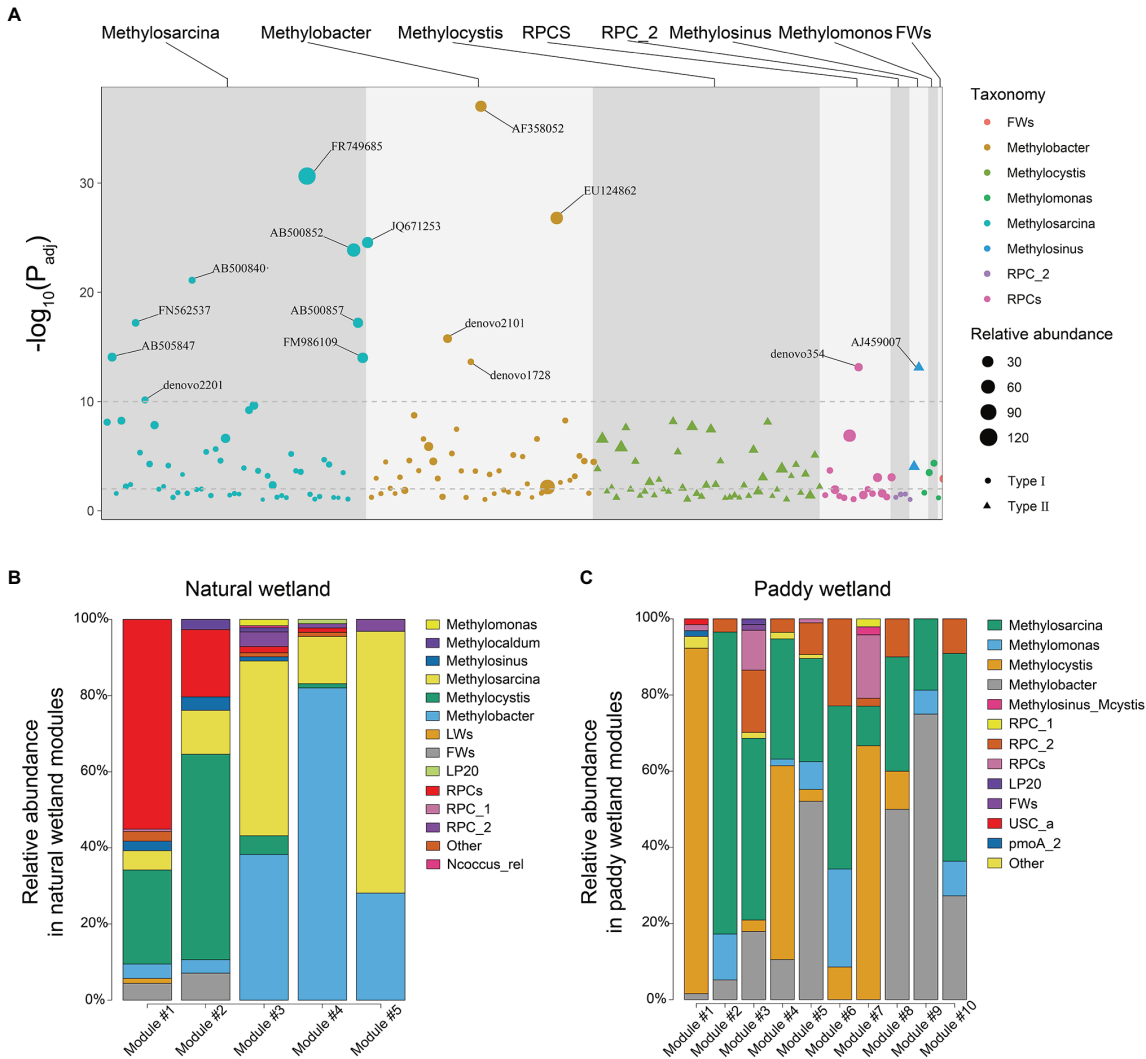
**(A)** Manhattan plots showing differential operational taxonomic units (OTUs) in networks of paddy and natural wetlands. OTUs that belonged to Type I were depicted as circles shape where Type II OTUs as triangle shape. The dashed line corresponded to the adjusted value of *p* threshold of significance (*α* = 0.01/0.001). The color of each dot represented the different taxonomic affiliation of the OTUs, and the size corresponded to their abundance in the respective samples. Gray backgrounds were used to denote the different taxonomic groups. **(B,C)** Relative abundance of the dominant genus in the respective main ecological modules of natural and paddy wetland networks, respectively.

Distinct patterns of co-occurrence relationships were observed between paddy and natural wetlands, which were determined based on the methanotrophic networks (Figure 4). A total of 1,416 positive and 124 negative links were observed in the network of paddy wetlands, whereas 3,127 positive and 366 negative links were detected in the network of natural wetlands, which involved a total of 464 and 576 OTUs, respectively (Supplementary Table S5). Degree scores defined as the number of direct associations of the involved node and betweenness centralities indicating the degree of the involved node passed by network paths significantly differed between paddy and natural wetlands (Degree scores: *F* = 26.68, *p* < 0.001; Betweenness centralities: *F* = 173.38, *p* < 0.001; Figure 4B). The network analysis suggested that network stability was higher for the natural wetland network than the paddy wetland network, as the former had a greater proportion of removed nodes (Figure 4C). We also compared the abundance of methanotrophs in the networks and found that type I methanotrophs were mainly composed of differentially abundant taxa (Figure 5A). Furthermore, network module analysis divided the paddy wetland network into 10 modules and the natural wetland network into 5 modules. The community composition of each module is shown in Figures 5B,C. Linear regression showed that modules 1, 3, 4, 5, and 7 in the network for paddy wetlands were significantly related to MOPs; modules 1, 2, and 5 in the network for natural wetlands were significantly related to MOPs (Supplementary Figure S5). Other topological properties, such as the clustering coefficient, average neighbors, and centralization, are shown in Supplementary Table S5.

The nodes in the networks were divided into network hubs, module hubs, connectors, and peripherals by calculating Zi and Pi values. A less hub-based and more connected network structure in both paddy and natural wetlands was observed (Supplementary Figure S6). There were 5 OTUs that were regarded as module hubs in paddy wetlands, which were most closely affiliated to *RPC_2* (1 OTU), *Methylosarcina* (1 OTU), *Methylobacter* (1 OTU), and *Methylocystis* (2 OTU). Seven module hubs belonged to *Methylosarcina* (2 OTUs), *Methylobacter* (3 OTUs), *RPCs* (1 OTU), and *Methylocystis* (1 OTU), all of which were type I with the exception of *Methylocystis* in natural wetlands (Supplementary Table S6).

### Assembly Processes of MOB Communities

**Figure 6:**
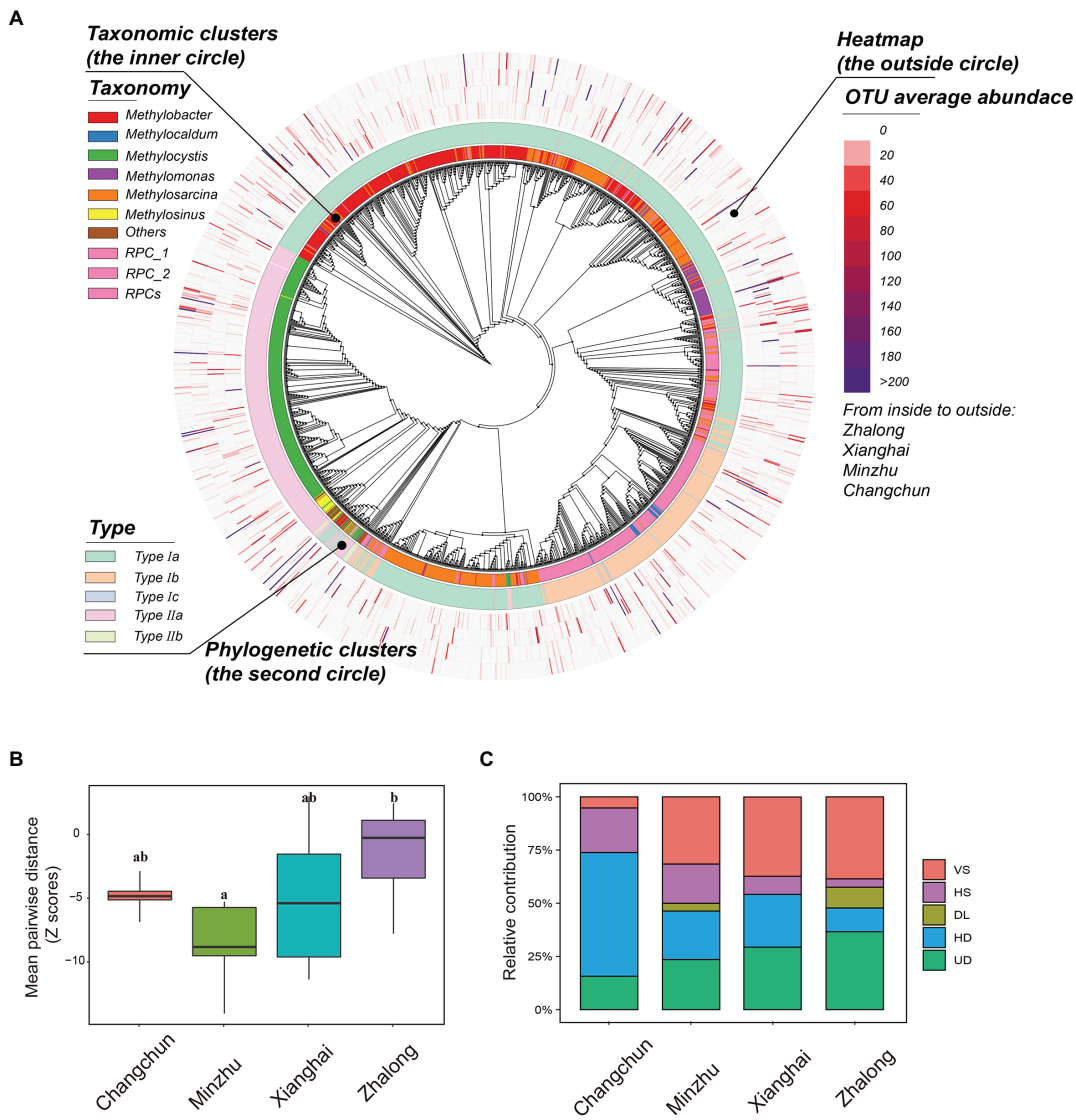
**(A)** Phylogenetic tree displaying the taxonomic information, phylogenetic clusters, and OTU average abundance from inside layer to the outside layer of active methanotrophic phylotypes in four wetland soils. **(B)** The standardized difference of mean pairwise distance for active methanotrophic communities in four wetland soils using standard deviation (Z-score). Boxes with different lowercase letters represent significant differences (*p* < 0.05). **(C)** The contributions of different assembly processes of active methanotrophic communities in four wetland soils. The five processes indicated the relative importance in dominating the community dissimilarity between two samples. VS, variable selection; HS, homogeneous selection; DL, dispersal limitation; HD, homogenizing dispersal; UD, undominated.

A phylogenetic tree was constructed to characterize the taxonomic distribution and relative abundance of methanotrophs (Figure 6A); the tree revealed that type I methanotrophs were the most common. Standardized MPD was calculated to characterize patterns of phylogenetic distance among methanotrophic communities (Figure 6B; Supplementary Table S7), and these calculations revealed clear variation in phylogenetic distance (among sites: *F* = 5.088, *p* = 0.005; among types: *F* = 5.458, *p* = 0.026). Analysis of community assembly processes (Figure 6C; Supplementary Table S8) revealed that the community turnover in CC was dominated by HD (~58.2%), followed by HS, UD, and VS. In MZ paddy wetlands, VS played an important role in community assembly (~31.6%), and UD, HD, and HS played equally important but secondary roles. The importance of stochastic processes in natural wetlands was highly variable compared with deterministic processes. Regression was used to evaluate the relationship between differences in MOPs and βNTI/RC_bray_ (Supplementary Figure S7). Differences in βNTI among paired sites were largely affected by VS (~28.2%) and negatively correlated with differences in MOPs. RC_bray_ was largely affected by DL (~3.37%) and UD (~26.3%) and positively correlated with differences in MOPs.

## Discussion

DNA-SIP experiments were conducted to study the structure profiling, co-occurrence relationships, and assembly processes of methanotrophic communities in paddy and natural wetlands. Paddy and natural wetlands are two important types of wetlands that play an important role in CH_4_ consumption driven by soil microorganisms. In our experiment, CH_4_ oxidation activity was high in all incubations from the two types of wetlands, and methanotrophic communities were successfully labeled by ^13^C; this permitted us to compare the structure and function of the active methanotroph community in both types of wetlands.

### Community Compositions

Methanotrophic communities were highly diverse, and the composition of these communities in paddy and natural wetlands was distinct. Four differentially abundant and indicative methanotrophs were detected through LEfSe analysis. *Methylobacter* was enriched in natural wetlands, known to consist of psychrophilic methanotrophs with cold-adapted properties (Wartiainen et al., 2006; Dieser et al., 2014). *Methylosarcina*, which was prevailing in paddy wetlands, widely occurs in human-made ecosystems (McDonald et al., 2008; Martineau et al., 2010). *FWs*, an uncultured phylogenetical cluster, are usually present in natural plant-rich wetlands (Zhu et al., 2007). *Methylosinus* consists of acidophilic bacteria and MOB (Zhang et al., 2020). These differentially abundant and indicative taxa explained a large amount of variation in community composition between paddy and natural wetlands.

### Co-occurrence Relationships

One major finding of our study was that the co-occurrence relationships of methanotrophic communities in paddy and natural wetlands were distinct (Figure 4). One explanation for this pattern might be attributed to the more phylogenetically diverse phenotypes in natural wetlands compared with paddy wetlands, given that the survival of microbes critically depends on biotic interactions (Faust and Raes, 2012; Segar et al., 2020). Previous studies have explored the relationships between network interactions and phylogenetic distance (Goberna et al., 2019). Positive interactions (aggregative links) tend to occur between phylogenetically different species, whereas negative interactions (segregative links) either occur because of niche similarities or fitness differences. Our findings were consistent with this hypothesis: average positive links (positive links per node) were higher in natural wetlands than in paddy wetlands. This suggests that communities with larger phylogenetic distances may be characterized by robust network relationships through cooperation and facilitation interactions. Alternatively, this pattern might be explained by the fact that natural wetlands are more oligotrophic than paddy wetlands; the soil organic matter and total nitrogen content were markedly lower in XH and ZL (Supplementary Table S1). Thus, the links between methanotrophic taxa were strong, which enhanced the resistance of the natural wetland communities to disturbance.

The microbial composition of network modules was complex, and the structure of the network modules in paddy and natural wetlands differed. Various functions in different modules of entire biomes have been reported in previous studies (Fan et al., 2018; Shi et al., 2019; Ma et al., 2020). Presumably, this finding can be explained by differences in the efficiency of CH_4_ consumption of methanotrophic guilds in different modules. Fan et al. (2019) showed that nitrogen fixation rates are significantly related to key modules of diazotrophic networks. The relative abundance of methanotrophs within different modules was correlated considerably with MOPs in this study (Supplementary Figure S5), indicating that module abundance and composition may modulate functional potentials (Bodelier et al., 2013). The niche partitioning of methanotrophs within a community can lead to the appearance of keystone species that dominate in a core function (e.g., module hubs) of CH_4_ consumption (Toju et al., 2018; Ma et al., 2020). Both rare and highly abundant methanotrophs occupied critical positions in the networks (Supplementary Figure S6). For example, the module hub *RPC_2* was rare in paddy wetlands. However, methanotrophs were only identified to the genus level, and most of their ecological and metabolic functions remain poorly known. Future work focusing on uncultured methanotrophs, such as the provisional genus *RPC_2*, is important for identifying the roles of these keystone guilds in wetland ecosystems.

### Assembly Processes

Community assembly processes that help us enhance the understanding of how communities were structured, also varied among different wetlands. Phylogenetic stochasticity was observed in ZL, and phylogenetic clustering was observed in XH, MZ, and CC (Figure 6B). Differences in functional potentials might be driven by phylogenetic stochasticity, but the efficiency of MOPs was limited in ZL. Saturated or flooding conditions (e.g., in paddy wetlands) were promoted by hydrologic mixing, and these conditions presumably enhanced the capacity of microorganisms to migrate across geographical regions (Liu et al., 2020), which might explain why the relative importance of HD and ecological drift was higher in paddy wetlands compared with other processes. Our findings were consistent with the results of Liu et al. (2020): UD contributed the most to shaping the communities in ZL natural wetlands, which enhanced the degree of phylogenetic stochasticity. By contrast, phylogenetic clustering can promote convergence in functional potentials and enhance the efficiency of MOPs depending on the methanotrophic guild. These results provide new insights into the CH_4_ consumption potentials of methanotrophs, especially for the large quantity of unculturable or unknown methanotrophic taxa.

The dominant process shifted from HD at high MOPs to VS at low MOPs (Figure 6C; Supplementary Table S8). This result indicates that the observed processes can drive patterns consistent with the findings of previous studies (Zhang et al., 2019; Liu et al., 2020). HD resulted in greater community similarity under intensive farm management in paddy wetlands than in natural wetlands. These findings validated the assembly theory of Stegen et al. (2015), in which HD is dominant in wetland ecosystems with weak environmental selection, possibly because of fertilization (e.g., Changchun paddy wetland). In contrast, wetlands in which environmental selection is stronger were dominated by VS (e.g., Zhalong natural wetland). Consistent with the results of Feng et al. (2018), fertilization induced a shift in the dominant process affecting the diazotrophic community from deterministic processes to stochastic processes, suggesting that DL and drift became increasingly important factors shaping communities compared with selection. Based on the correlation between the assembly processes of methanotrophs and differences in the CH_4_ oxidation potential, dispersal limitation and ecological drift were strongly associated with the potentials, explaining 9.0 and 13.7% of variations, respectively. These results indicated that the functional potential might be closely related to stochastic processes. Exploration of the associations between processes and functions is novel and adventurous, and this involves how to define and determine phylogenetic distance/community dissimilarity and the relative magnitude of different functional parameters in the future.

## Conclusion

In sum, the co-occurrence relationships and assembly processes of methanotrophic communities in paddy and natural wetlands in Northeast China were distinct. DNA-SIP experiment and high-throughput sequencing were used to compare the active methanotrophic communities between paddy and natural wetlands; network and phylogenetic analysis were applied to reveal co-occurrence relationships and assembly processes. Analysis of differences in the community composition revealed that *Methylosarcina*, *Methylobacter*, *Methylosinus*, and *FWs* were the most differentially indicative taxa between paddy and natural wetlands, and these taxa potentially contribute to differences in CH_4_ consumption. The network structure of methanotrophic communities might be more stable in natural wetlands than in paddy wetlands. Degree scores and betweenness centralities differed between paddy and natural wetlands. Exploration of the relationships between module abundance and MOP revealed that the relative abundance of key sub-communities within modules accounted for the observed variation in functional potentials. Although stochastic processes contributed the most to community turnover, additional work is needed to credibly explore the potential links between community turnover and functional differences. The results of this study enhance our understanding of differences in community profiling, the co-occurrence relationships, and assembly processes of active methanotrophic communities between paddy and natural wetlands.

## Data Availability Statement

The data sets presented in this study can be found in online repositories. The names of the repository/repositories and accession number(s) can be found in the article/Supplementary Material.

## Author Contributions

All authors contributed the intellectual input and assistance to this study and manuscript preparation. HC, YS, JL, and XL provided initial concept and analysis. XL, LZ, RL, RX, and CL carried out field sampling, soil microbial, and chemical analyses. XL, TY, G-FG, and HC wrote manuscript. All authors contributed to the article and approved the submitted version.

## Funding

This work was supported by the Strategic Priority Research Program of Chinese Academy of Sciences (XDA28020202), the National Natural Science Foundation of China (91951109), and the Second Tibetan Plateau Scientific Expedition and Research Program (STEP, 2019QZKK0503).

## Conflict of Interest

The authors declare that the research was conducted in the absence of any commercial or financial relationships that could be construed as a potential conflict of interest.

## Publisher’s Note

All claims expressed in this article are solely those of the authors and do not necessarily represent those of their affiliated organizations, or those of the publisher, the editors and the reviewers. Any product that may be evaluated in this article, or claim that may be made by its manufacturer, is not guaranteed or endorsed by the publisher.

## Footnotes

^1^ https://github.com/rdpstaff/fungene_pipeline

^2^ https://gephi.org/

^3^ https://www.r-project.org
