# Supplementary material for: Distinct Co-occurrence Relationships and Assembly Processes of Active Methane-Oxidizing Bacterial Communities Between Paddy and Natural Wetlands of Northeast China
Source: Front Microbiol. 2022 Jan 26;13:809074. doi: 10.3389/fmicb.2022.809074 (PMC8826055; doi:10.3389/fmicb.2022.809074)
Supplement: Supplementary file 1 [file Table_1.DOCX]

**Supplementary Materials**

Distinct co-occurrence relationships and assembly processes of active methane-oxidizing bacterial communities between paddy and natural wetlands of Northeast China

Xu LIU^1,2^, Yu SHI^3^, Teng YANG^1,2^, Guifeng GAO^1,2^, Liyan ZHANG^4^, Ruoyu XU^5^, Chenxin LI^5^, Ruiyang LIU^5^, Junjie LIU^6^, Haiyan CHU^1,2, *^

1 State Key Laboratory of Soil and Sustainable Agriculture, Institute of Soil Science, Chinese Academy of Sciences, Nanjing 210008 (China)

2 University of Chinese Academy of Sciences, Beijing 100049 (China)

3 State Key Laboratory of Crop Stress Adaptation and Improvement, School of Life Sciences, Henan University, Kaifeng 475004 (China)

4 Key Laboratory of Integrated Regulation and Resource Development on Shallow Lake of Ministry of Education, College of Environment, Hohai University, Nanjing 210098 (China)

5 High School Affiliated to Nanjing Normal University, Nanjing 210003 (China)

6 Key Laboratory of Mollisols Agroecology, Northeast Institute of Geography and Agroecology, Chinese Academy of Sciences, Harbin 130102 (China)

Corresponding Author:

Haiyan CHU*

Institute of Soil Science, Chinese Academy of Sciences, 71 East Beijing Road, Nanjing

Email address: hychu@issas.ac.cn

Figure S1. Quantitative distributions of *pmoA* gene copy numbers across the entire buoyant density gradient of the fractionated DNA samples. Green background represented light layers incubated by ^12^C-labeled methane; Orange background represented heavy layers incubated by ^13^C-labeled methane. The horizontal axis was proportion of *pmoA* gene copy numbers, the vertical axis was different fractionations.


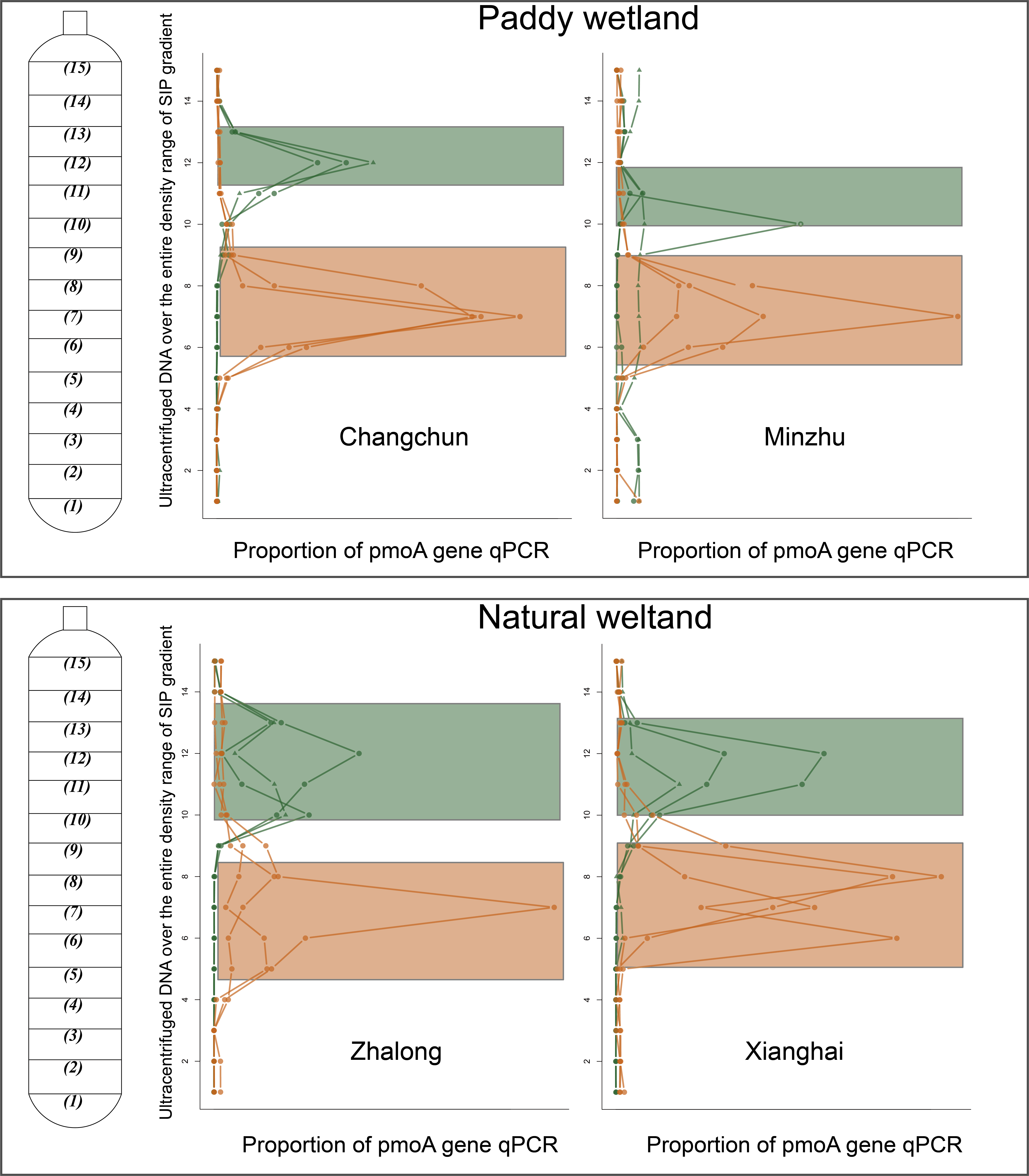


Figure S2. (A) Relative abundance of ^12^C-labeled MOB on genus levels, identified with *pmoA* gene sequencing in four wetland soils. (B) Principal coordinates analysis (PCoA) biplots of Bray-Curtis distance for the ^12^C-labeled methanotrophic communities of four wetland soils.


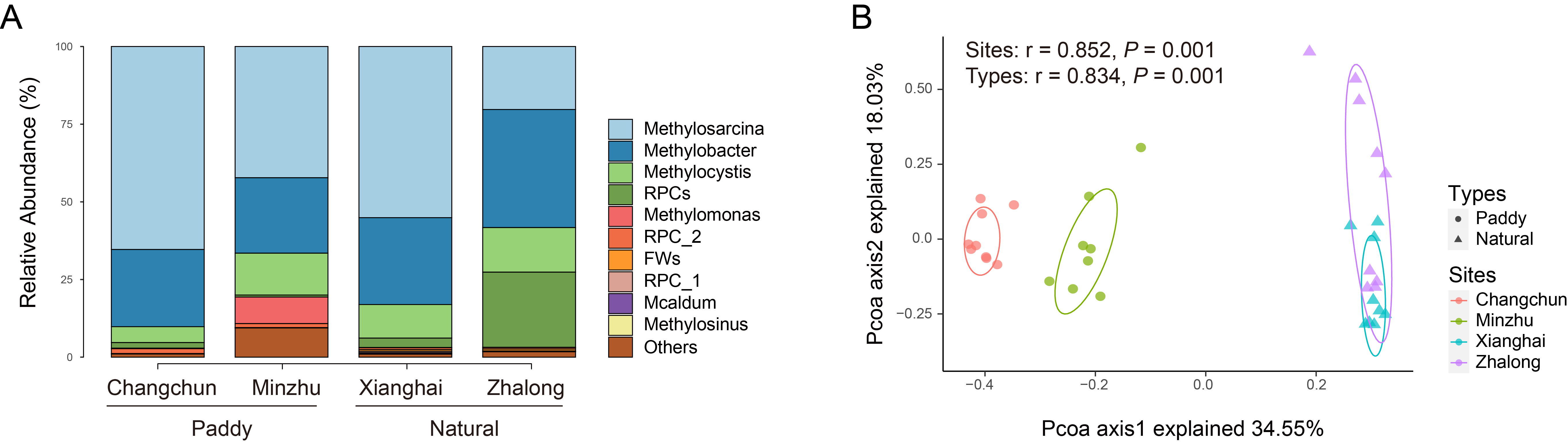


Figure S3. Faith’s Phylogenetic Diversity (A) and Species richness (B) of active methanotrophic communities in four wetlands. LSD test was used to multi-group comparison.


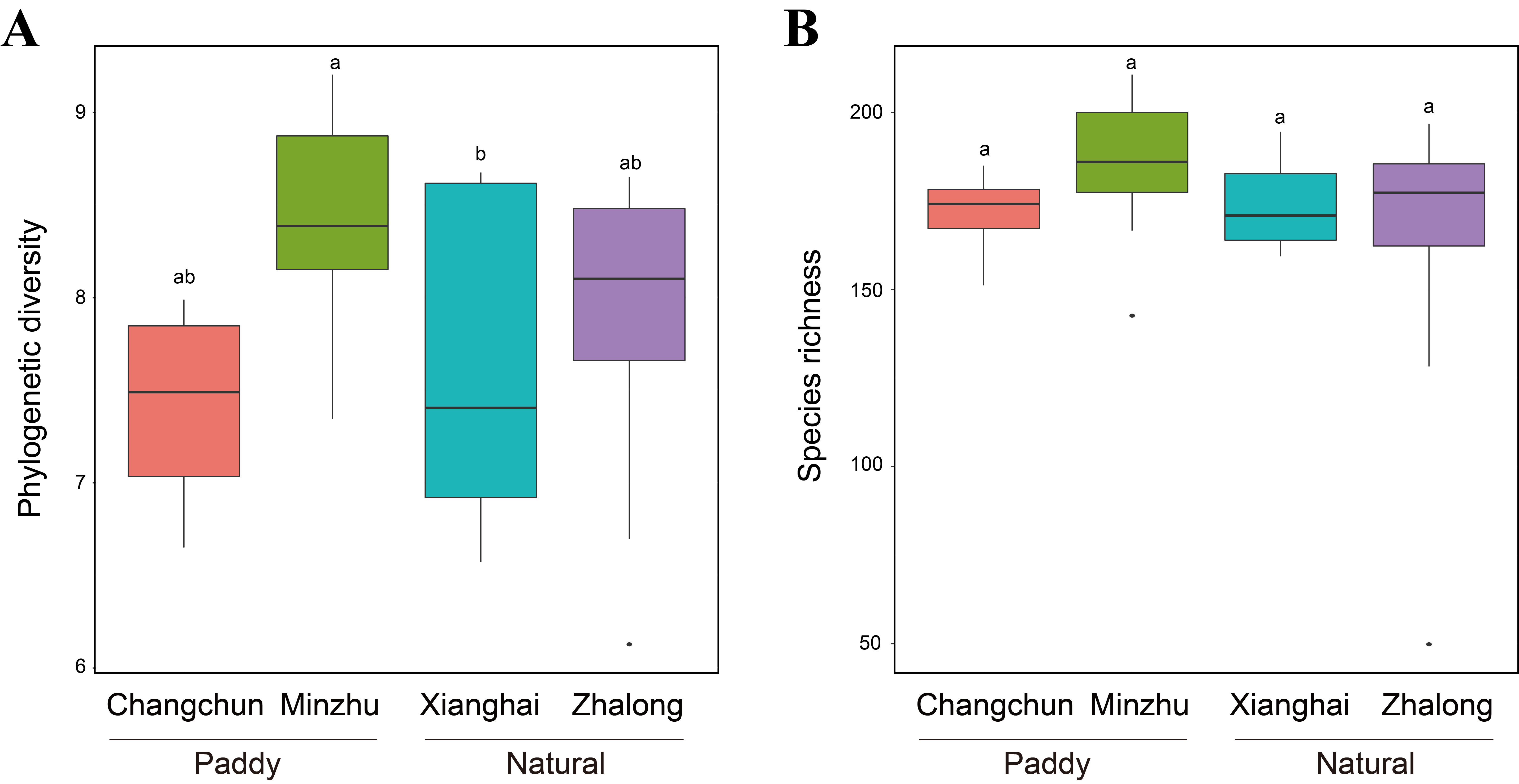


Figure S4. Taxon-specific threshold indicator taxa analysis with the gradient of MOPs in paddy and natural wetlands, showing significant indicator taxa (*P* ≤ 0.05). Z^+^ and Z^−^ taxa increased or decreased in the frequency of occurrence and abundance with MOPs, respectively. The solid circle represented Z^−^ taxa and the hollow circle represented Z^+^ taxa. Blue affiliation represented type Ⅰ methanotrophs and red affiliations represented type Ⅱ methanotrophs.


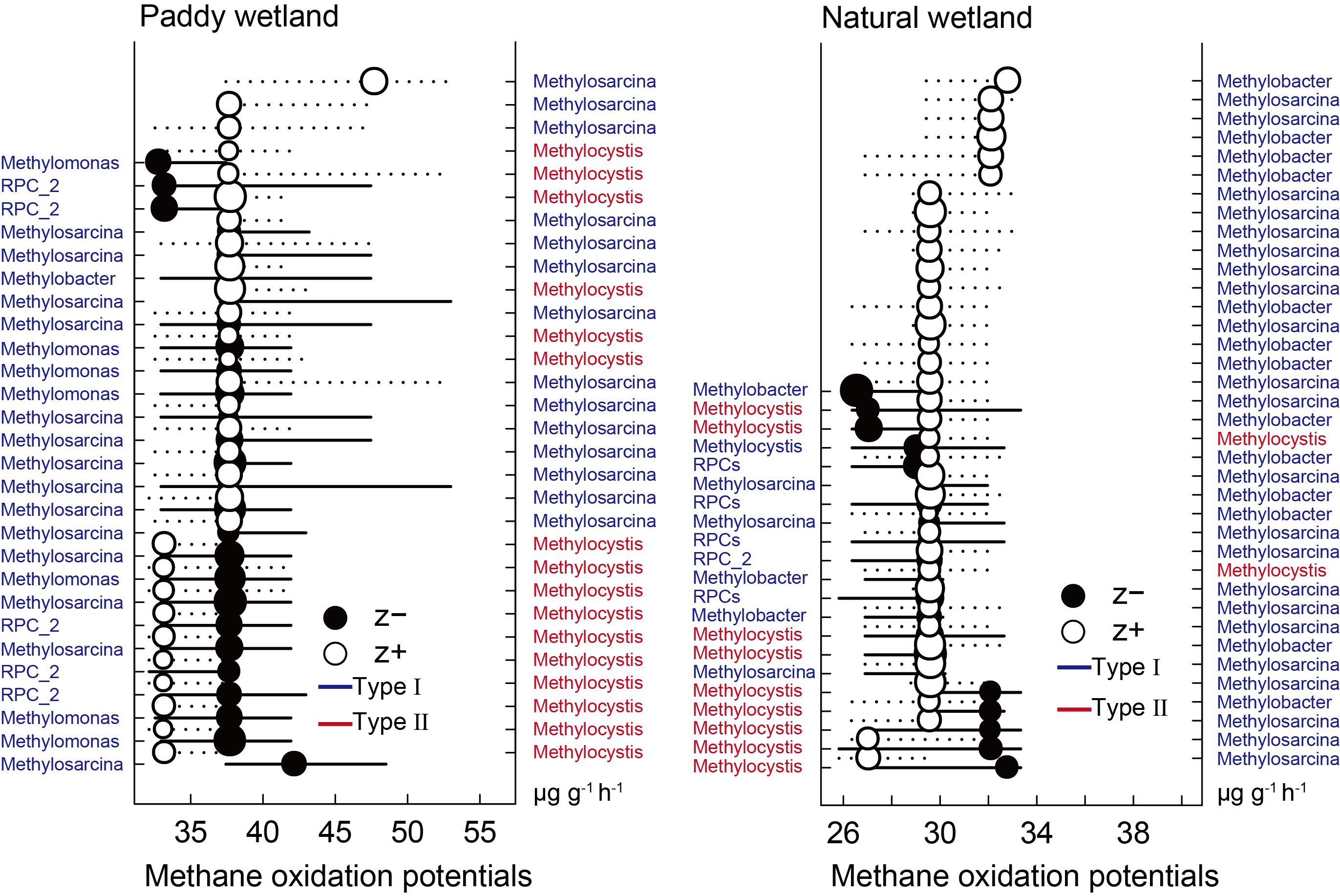


Figure S5. The regression relationships between methane oxidation potentials and module abundance in natural wetland ecosystems (A) and paddy wetland ecosystems (B). Module abundance were calculated by Z-score standardization of relative abundance of active methanotrophs.


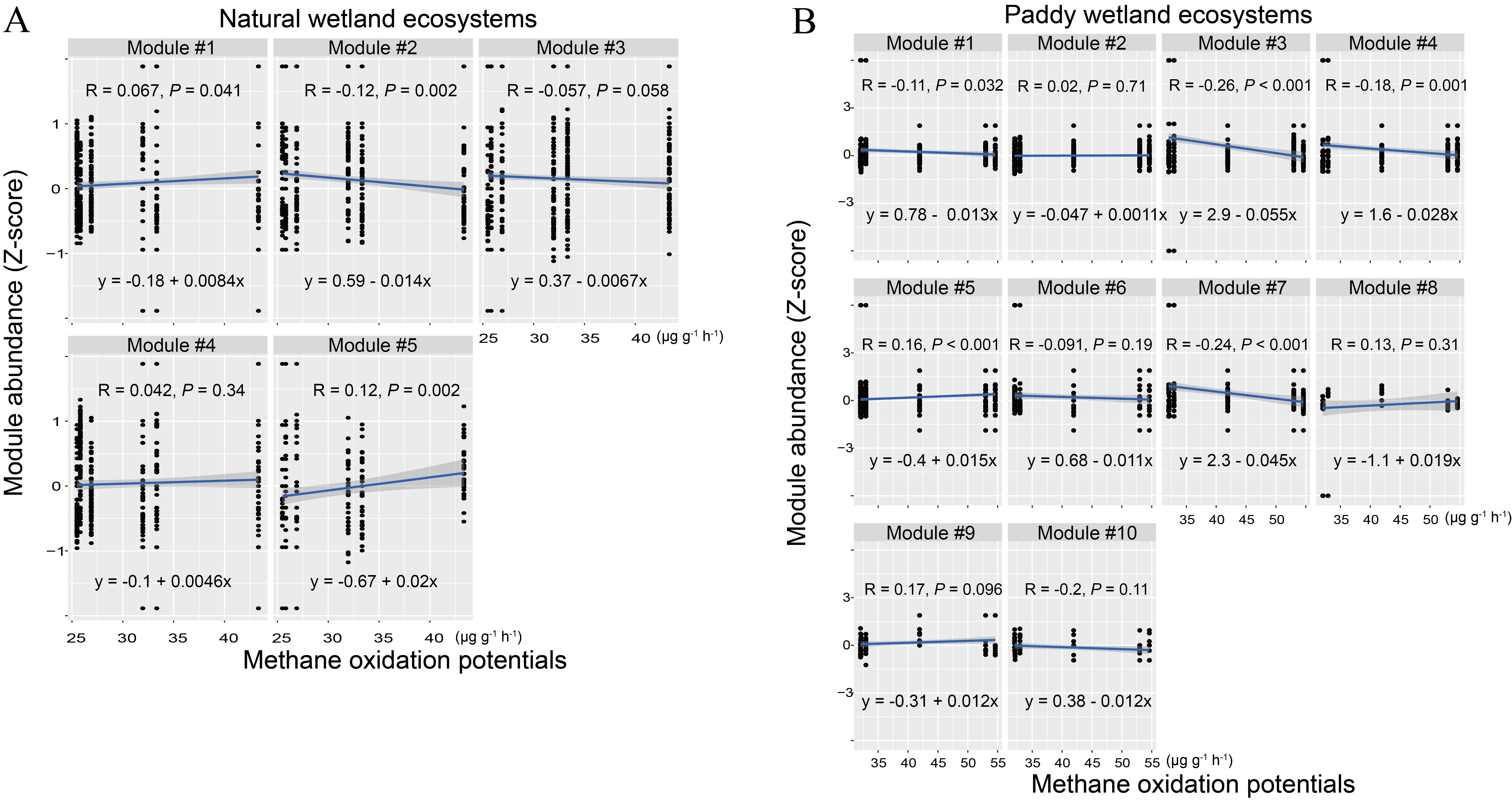


Figure S6. Zi-Pi plots showing distributions of OTUs based on their topological roles in co-occurrence networks. Zi on behalf of within-module connectivities and Pi on behalf of among-module connectivities. Threshold values of Zi and Pi for categorizing OTUs were 2.5 and 0.62, respectively.


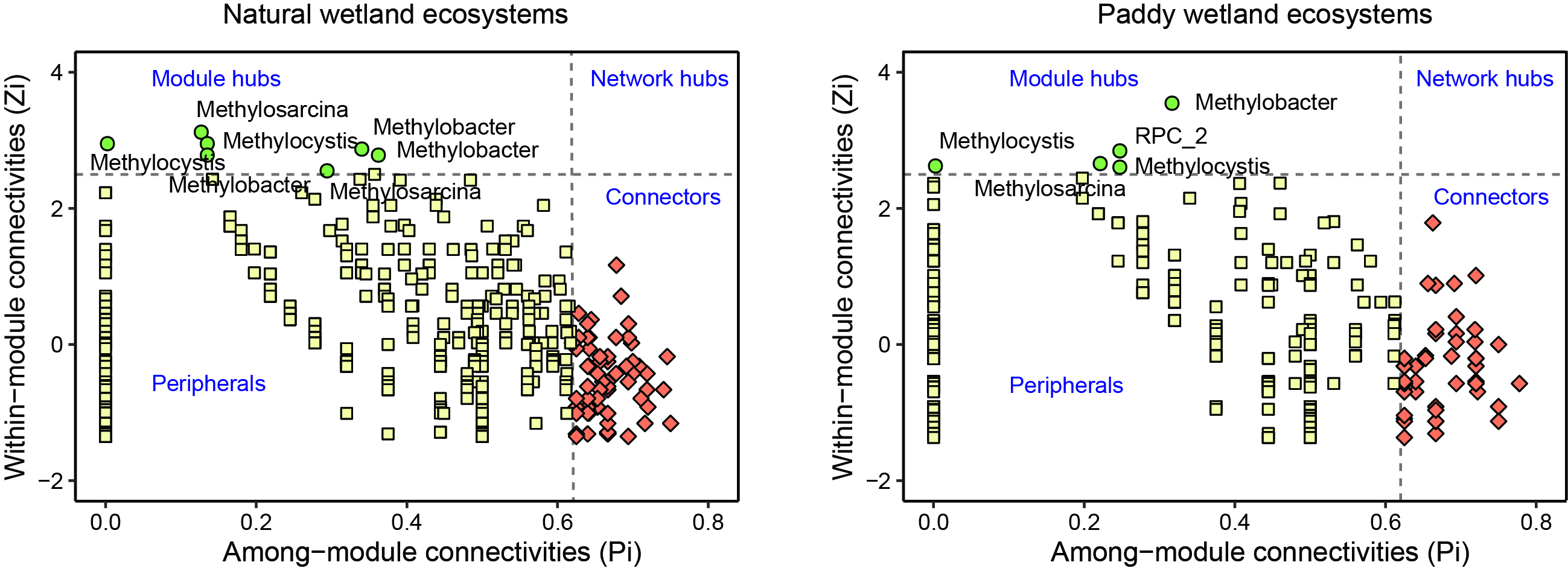


Figure S7. The regression relationships between the difference of methane oxidation potentials (ΔMOPs) and βNTI/RC_bray_. The processes of variable selection and homogenizing dispersal which were significant were shown. RC_bray_ represented Raup–Crick_bray-curtis_ dissimilarity. βNTI represented β-nearest taxon index.


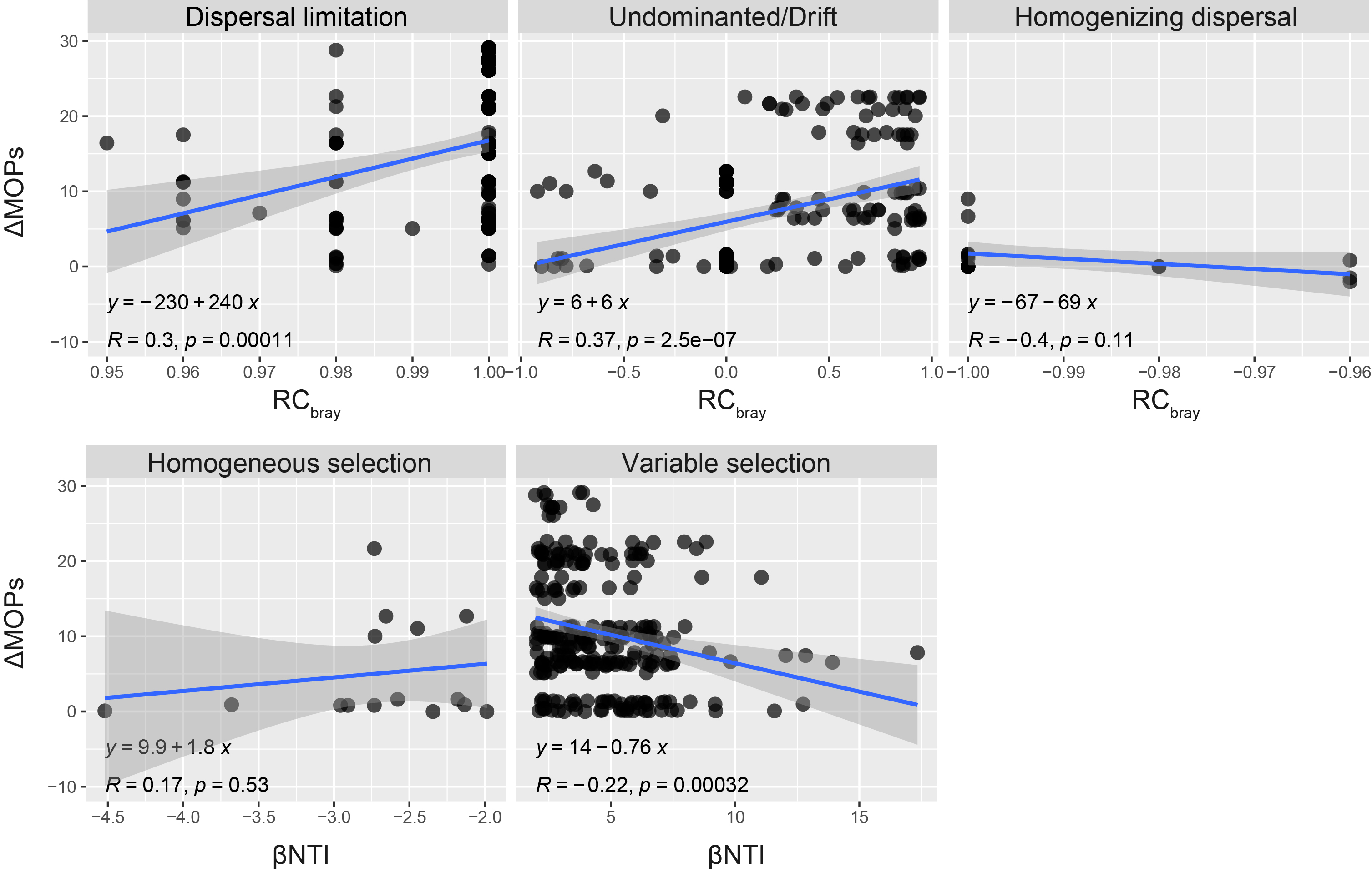


Table S1. Basic information of four typical wetlands in Northeast China. Lon&Lat represented Longitude and latitude; MAT represented mean annual temperature; MAP represented mean annual precipitation; SOM represented soil organic matters; TN represented total nitrogen.

| **Sites** | **Type** | **City** | **Main** | **Lon&Lat** | **MAT** | **MAP** | **pH** | **SOM** | **TN** |
| --- | --- | --- | --- | --- | --- | --- | --- | --- | --- |
| **/** | **/** | **/** | **vegetation** | **°** | ℃ | **mm** | **/** | **g/kg** | **g/kg** |
| Zhalong | Natural wetland | Qiqihar | *Phragmuses cmmunis*; *Carex* | 7.19 N 124.24 E | 3.5 | 416 | 8.88 | 6.1 | 0.2 |
| Xianghai | Natural wetland | Baicheng | *Phragmuses cmmunis* | 45.04 N 122.37 E | 5.1 | 408 | 9.21 | 11.9 | 0.6 |
| Minzhu | Paddy wetland | Harbin | *Rice*;  *Corn* | 45.86 N 126.81 E | 3.5 | 530 | 6.05 | 32.5 | 3.4 |
| Changchun | Paddy wetland | Changchun | *Rice*;  *Corn* | 3.75 N 125.25 E | 4.6 | 530 | 6.8 | 21.5 | 4.1 |

Table S2. The Spearman correlations between MOPs and alpha diversity.

| **Correlations** | **Natural wetlands** | | **Paddy wetlands** | |
| --- | --- | --- | --- | --- |
|  | r | *P* | r | *P* |
| Species Richness | 0.143 | 0.787 | -0.6 | 0.208 |
| Phylogenetic diversity | -0.029 | 0.957 | -0.714 | 0.111 |

Table S3. The Spearman correlations between MOPs and relative abundance of dominant methane-oxidizing bacterial genus.

| **Correlations** | **Methane oxidation potentials** | |
| --- | --- | --- |
|  | r | *P* |
| Methylosarcina | 0.734 | 0.007 |
| Methylobacter | -0.406 | 0.191 |
| FWs | -0.571 | 0.052 |
| Methylosinus | -0.397 | 0.202 |
| Methylocystis | 0.308 | 0.331 |
| RPCS | -0.434 | 0.159 |

Table S4. Analysis of similarities (ANOSIM) of active methanotrophic communities between wetland types and between wetland sites, based on Bray-Curtis, Weighted Unifrac, and Unweighted Unifrac distance.

| **Distance** | **Wetland Types** | | **Wetland Sites** | |
| --- | --- | --- | --- | --- |
|  | r | *P* | r | *P* |
| Bray-Curtis distance | 0.842 | 0.001 | 0.896 | 0.001 |
| Weighted-Unifrac distance | 0.416 | 0.001 | 0.483 | 0.001 |
| Unweighted-Unifrac distance | 0.893 | 0.001 | 0.882 | 0.001 |

Table S5. The network topological properties of co-occurrence networks. Subnetworks included four wetland sites and two wetland types.

| **Network** | **Paddy** | **Natural** | **Changchun** | **Minzhu** | **Xianghai** | **Zhalong** |
| --- | --- | --- | --- | --- | --- | --- |
| Positive link | 1416 | 3127 | 254 | 336 | 868 | 1029 |
| Negative link | 124 | 366 | 89 | 62 | 216 | 209 |
| Total link | 1540 | 3493 | 343 | 398 | 1084 | 1238 |
| Mean degree | 6.624 | 12.130 | 2.349 | 2.690 | 6.590 | 6.674 |
| Modularity | 0.519 | 0.414 | 0.732 | 0.702 | 0.430 | 0.473 |
| Clustering coefficient | 0.108 | 0.110 | 0.091 | 0.069 | 0.104 | 0.093 |
| Betweenness centrality | 0.006 | 0.003 | 0.059 | 0.028 | 0.007 | 0.008 |

Table S6. Network topological and taxonomic information for the module hubs in co-occurrence networks of paddy and natural wetlands.

| **ID** | **Role** | **Taxonomy** | **Type** | **Pi** | **Zi** | **Module** |
| --- | --- | --- | --- | --- | --- | --- |
| Paddy |  |  |  |  |  |  |
| FN649541 | module hub | RPC_2 | Type Ⅰa | 0.245 | 2.832 | 6 |
| denovo894 | module hub | Methylosarcina | Type Ⅰa | 0.219 | 2.651 | 4 |
| FM986175 | module hub | Methylocystis | Type Ⅱa | 0.000 | 2.613 | 1 |
| AM849619 | module hub | Methylocystis | Type Ⅱa | 0.245 | 2.600 | 7 |
| AB064369 | module hub | Methylobacter | Type Ⅰa | 0.314 | 3.537 | 5 |
| Natural |  |  |  |  |  |  |
| denovo1129 | module hub | Methylosarcina | Type Ⅰa | 0.292 | 2.544 | 5 |
| denovo3092 | module hub | Methylobacter | Type Ⅰa | 0.133 | 2.770 | 3 |
| EF625908 | module hub | Methylobacter | Type Ⅰa | 0.360 | 2.770 | 3 |
| EF623673 | module hub | Methylobacter | Type Ⅰa | 0.338 | 2.866 | 4 |
| denovo1512 | module hub | RPCs | Type Ⅰb | 0.133 | 2.939 | 1 |
| EU131056 | module hub | Methylocystis | Type Ⅱa | 0.000 | 2.939 | 1 |
| denovo3091 | module hub | Methylosarcina | Type Ⅰa | 0.124 | 3.113 | 3 |

Table S7. Mean pairwise distance of different incubated samples. ntaxa represented the numbers of active methanotrophic OTUs; mpd.obs represented mean pairwise distance value in true models; mpd.rand.mean represented mean values of mean pairwise distance in random models; mpd.rand.sd represented standard deviance of mean pairwise distance in random models; mpd.obs.z represented z scores of mean pairwise distance value in true models; mpd.obs.p represented *P* values of mean pairwise distance value in true models.

| **Site** | **Zhalong** | **Xianghai** | **Minzhu** | **Changchun** |
| --- | --- | --- | --- | --- |
| ntaxa | 168 | 173 | 180 | 168 |
| mpd.obs | 0.70 | 0.61 | 0.56 | 0.63 |
| mpd.rand.mean | 0.74 | 0.74 | 0.74 | 0.74 |
| mpd.rand.sd | 0.02 | 0.02 | 0.02 | 0.02 |
| mpd.obs.z | -1.70 | -5.61 | -8.50 | -4.93 |
| mpd.obs.p | 0.01 | 0.01 | 0.01 | 0.01 |
| Type | Natural | Natural | Paddy | Paddy |

Table S8. The relative importance of ecological assembly processes within four wetland sites.

| **Process** | **Type** | **Zhalong** | **Xianghai** | **Minzhu** | **Changchun** |
| --- | --- | --- | --- | --- | --- |
| Variable Selection | deterministic | 38.6% | 37.3% | 31.6% | 5.2% |
| Homogeneous Selection | deterministic | 3.9% | 8.5% | 18.4% | 20.9% |
| Dispersal Limitation | stochastic | 9.8% | 0.0% | 3.7% | 0.0% |
| Homogenizing Dispersal | stochastic | 11.1% | 24.8% | 22.8% | 58.2% |
| Undominated/drift | stochastic | 36.6% | 29.4% | 23.5% | 15.7% |
